# Subjective Cognitive Decline, Inter‐Personal Attachment Style and Relationship Quality

**DOI:** 10.1002/jgf2.70076

**Published:** 2025-10-26

**Authors:** Mohamed Eshmawey, Sonja M. Kagerer, Federica Ribaldi, Aïda B. Fall, Paul G. Unschuld

**Affiliations:** ^1^ Department of Geriatric Psychiatry Geneva University Hospitals Geneva Switzerland; ^2^ Memory Center, Department of Geriatrics Geneva University Hospitals Geneva Switzerland; ^3^ Departement of Psychiatry Geneva University Hospitals Geneva Switzerland; ^4^ Departement of Geriatric Psychiatry University Hospital of Psychiatry Zurich Switzerland; ^5^ Faculty of Medicine Geneva University Geneva Switzerland

**Keywords:** attachment style, close relationship, couple life, subjective cognitive decline

## Abstract

**Background:**

Subjective cognitive decline (SCD) is characterized by the perception of cognitive dysfunction, and it could be one of the early signs of dementia. While SCD is a common phenomenon in old persons, little is known about how it affects interpersonal relationships.

**Methods:**

We conducted a cross‐sectional study involving 16 patients with SCD and 39 volunteers recruited from the COSCODE study. The Hazan and Shafer questionnaires were used to assess patients' attachment styles. The Experience in Close Relationships Scale was used to assess individual differences in attachment‐related anxiety and attachment‐related avoidance. A Wilcoxon rank‐sum test was performed to test for differences between groups, and *p*‐values were Bonferroni‐corrected.

**Results:**

SCD is associated with lower disorganized attachment scores (*p* = 0.01). SCD was not associated with experience in close relationships.

**Conclusion:**

Coping with progressive cognitive decline is a difficult mission. The results of our study on persons with SCD help us to better understand changes in couple relationships before the onset of dementia.

## 1 Background

Individuals with subjective cognitive decline (SCD) have the perception of cognitive problems without showing objective neuropsychological findings on cognitive tests [1]. At this early stage, these patients still have sufficiently intact cognitive performance that can be directed toward compensation of function [2, 3]. SCD is a frequent finding in the elderly, and it is closely related to the risk of old age‐related cognitive impairment and Alzheimer's disease (AD) [4, 5]. Recent studies identified several psychological and behavioral factors associated with SCD, including maintained professional activity, anxiety‐related personality traits, and depressive psychopathology [5, 6]. Various studies have linked SCD to biological markers of Alzheimer's disease and brain degeneration. Recently, longitudinal studies suggested an association between SCD and increased risk of future objectively measurable cognitive impairment [7].

One of the goals of cognitive disorder treatment is to improve the quality of life for both patients and their partners. The partner must balance the additional role of caregiver with the original role of an intimate partner [8, 9]. A growing number of studies show that close relationships and intimacy remain important for patients suffering from cognitive disorders [10, 11]. Cognitive decline has a significant impact on the relationships of couples [12]. This decline in cognitive abilities, and the fear of further deterioration, directly affects the dynamics between life partners [13]. Receiving and providing care related to cognitive decline affects couples' lives and may result in changing patterns of intimate relationships [14]. Changes in responsibilities, roles, and self‐esteem have a significant impact on couple relationships [15]. Cognitive decline and behavioral changes such as decision‐making, reward recognition, emotional contagion, empathy, and motivation may contribute to decreased relationship satisfaction [8, 16].

Despite its importance, the quality of social life and interpersonal relationships in people with cognitive impairment is rarely assessed in academic studies and, to our knowledge, is not an outcome measure in any current clinical trial. In particular, to our knowledge, there has been no published research examining relationship quality in the context of SCD, despite the suggested association between SCD and increased risk of Alzheimer's disease. We conducted an observational study to assess attachment styles and perceived relationship quality in people with SCD.

## 2 Methods

This study was approved by the local Ethics Committee of Geneva University Hospitals, and all participants provided written informed consent according to the Declaration of Helsinki.

### 2.1 Study Design

We performed a cross‐sectional study involving 16 patients suffering from SCD and 39 healthy volunteers. Participants were recruited as a part of a larger study (COSCODE) that focus on interpreting signs and symptoms associated with different etiologies of SCD [17]. COSCODE is an observational, longitudinal (4 years), prospective clinical cohort study involving 120 SCD, and 80 control study participants (40 healthy control and 40 mild cognitive impairment participants). The diagnosis of SCD is based on the following criteria: persons asking for help and consulting to the Geneva Memory Center for self‐experience of deterioration in cognitive abilities, without objective cognitive impairment detected through formal neuropsychological testing. The negative control group was recruited by an advertising campaign of the memory center of the Geneva University hospitals.

General inclusion criteria of the COSCODE study are the following: (1) age over 40 years, (2) no major psychiatric disorders, (3) no severe behavioral disturbances (e.g., ability to undergo the procedures of the study), (4) no severe systemic diseases (e.g., renal failure, heart failure, decompensated diabetes), and (5) no severe diseases such as a malignant neoplasm within the last 5 years or other life‐threatening conditions. Patients suffering from mild stable depression who could undergo the procedures of the study were included in the research. Also, patients with stable mild to moderate renal or cardiac chronic diseases were included in the study.

For the current study, only SCD participants and healthy volunteers from the control group were included, who had filled in the Hazan and Shafer and the Experience in Close Relationships‐Revised questionnaire at the time of data collection.

### 2.2 Scales

*Hazan and Shafer questionnaire* used to assess patients for attachment styles [18]. Bowlby and Ainsworth concluded that there were three major styles of attachment: secure attachment, ambivalent‐insecure attachment, and avoidant‐insecure attachment [19]. In 1986, Main and Solomon added a fourth attachment style known as disorganized‐insecure attachment [20]. In 1987, Hazan and Shaver explored the possibility that couple relationships are an attachment process. They consider this as a biosocial process by which romantic love is formed between adults, just as childhood relationships are formed with parents [18]. Attachment styles are used to describe patterns of attachment in close relationships. These attachment styles are centered on how partners interact [21].

This self‐report scale is designed to assess adult attachment within a four‐category framework. Style A corresponds to secure, B corresponds to avoidant‐insecure, C corresponds to ambivalent‐insecure, and style D corresponds to disorganized‐insecure attachment patterns. On a scale of 0 to 100, the patient determines how many percentages he belongs to the variant.

*Experience in close relationships‐revised (ECR‐R) questionnaire* used to assess individual differences with respect to attachment‐related anxiety (i.e., the extent to which people are insecure vs. secure about the availability and responsiveness of romantic partners) and attachment‐related avoidance. ECR‐R is a commonly used self‐reporting questionnaire used to measure adult attachment styles [22].

A total of 36 items comprise the attachment‐related anxiety scale and the attachment‐related avoidance scale. The order in which these items are presented is randomized. Each item is rated on a 7‐point scale where 1 = strongly disagree and 7 = strongly agree. For each patient, we calculated the average of scores for all items within each scale. ECR‐R is designed to assess a general pattern of adult attachment and is helpful in conceptualizing how patients respond to romantic relationships. Results of ECR‐R consist of two scores: attachment anxiety and attachment avoidance.

### 2.3 MMSE

The memory center of Geneva University hospitals is authorized to use the mini‐mental state examination (MMSE) consensual French version of GRECO Consortium.

### 2.4 Types of Attachment Styles

#### 2.4.1 Secure

It is the most common attachment style. Individuals with this style feel safe and protected in their relationships. They are able to establish long‐lasting relationships and express their feelings with ease.

#### 2.4.2 Avoidant‐Insecure

Individuals with this style of attachment use distancing strategies in their relationships. They value independence, withdraw from emotional closeness, have a negative view of others, and focus on their own needs.

#### 2.4.3 Ambivalent‐Anxious‐Insecure

Individuals with this attachment style often display traits such asstruggling with fear of abandonment, feeling threatened by a partner's autonomy, seeking constant reassurance, and being uncomfortable with perceived distance within the relationship.

#### 2.4.4 Disorganized

This attachment style is characterized by conflicting emotions and behaviors. Individuals with this style experience difficulties in trusting partners, and they feel conflicted about how to behave in couple life. They often have difficulties maintaining healthy relationships because of their contradictory emotions [19, 20, 21].

### 2.5 Statistical Methods

Data were fed to the computer and analyzed using IBM SPSS software package version 20.0 (Armonk, NY: IBM Corp) and MATLAB (Version 2025a, Statistics and Machine Learning Toolbox Version 25.1) software. Categorical data were represented as numbers and percentages. Distributed data were expressed as range (minimum and maximum), mean, standard deviation, and median. Wilcoxon rank‐sum test was used to compare differences between groups. Bonferroni correction was applied to correct for multiple testing. Univariate and multivariate linear regression analyses were used to detect the effect of SCD on attachment score. The following covariates were included in the regression analyses based on their potential relevance to attachment style: age, MMSE score, years of education, and presence of alcohol consumption. Significance of the obtained results was judged at the 5% level.

## 3 Results

Demographic and clinical characteristics of the SCD patients and volunteers are displayed in Table 1.

**TABLE 1:** sociodemographic and clinical features of the study groups.

	SCD (*n* = 16)	Volunteer (*n* = 39)	*p*
Age (years)			
Mean ± SD	62.9 ± 8.1	62 ± 7.2	0.60
Median (min–max)	62 (48–77)	62 (50–79)	
Gender			
Male	3 (18.8%)	7 (17.9%)	0.96
Female	13 (81.3%)	32 (82.1%)	
Education (years)			
Mean ± SD	15.3 ± 3	15 ± 3	0.62
Median (min–max)	16 (9–19)	15 (10–24)	
MMSE score			
Mean ± SD	29 ± 3	28.8 ± 1	0.59
Median (min–max)	29 (27–30)	29 (26–30)	
Medical history			
Depression	2 (12.5%)	5 (12.8%)	0.98
Neurological	3 (18.8%)	4 (10.3%)	0.40
Cardiovascular	3 (18.8%)	1 (2.6%)	0.04*
Hyperchol	5 (31.3%)	3 (7.7%)	0.03*
Hypertension	3 (18.8%)	2 (5.1%)	0.12
Respiratory	1 (6.3%)	3 (7.7%)	0.87
Hepatic	1 (6.3%)	1 (2.6%)	0.53
Renal	3 (18.8%)	0 (0%)	0.01*
Alcohol	3 (18.8%)	2 (5.1%)	0.12
Cancer	2 (12.5%)	1 (2.6%)	0.15

*Note: p*: *p* value for comparing between the studied groups (Wilcoxon rank‐sum test).

Abbreviation: SD, standard deviation.

* Statistically significant at *p* ≤ 0.05.

The mean age of the participants was 62.9 years (SD 8.1), and the majority (81.3%) were female. Distribution of studied cases according to diagnosis (*n* = 55): 16 patients (29.1%), 39 volunteers (70.9%). SCD was correlated with hypercholesterolemia, a medical history of cardiovascular and renal diseases. SCD was not associated with age. No association was found between SCD and a history of depression.

The presence of SCD was associated with lower disorganized attachment style scores (*p* = 0.002, *p* (Bonferroni corrected) = 0.01) (SCD group: mean 0.8, SD 2.6, Control group: mean 16.2, SD 22.4). In the univariate linear regression analysis, the presence of SCD was significantly associated with lower disorganized attachment style scores (*B* = −15.4, 95% CI: −27.1 to −3.7; *p* = 0.011). None of the other covariates (age: *p* = 0.995, MMSE: *p* = 0.908, years of education: *p* = 0.494, alcohol consumption: *p* = 0.209) showed a significant association with disorganized attachment style in the univariate analysis. This association remained significant after adjustment for age, MMSE score, years of education, and alcohol consumption (*B* = −14.4, 95% CI: −26.9 to −1.9; *p* = 0.025) (Tables 2 and 3). SCD was not associated with experience in close relationships. We did not find that SCD was related to significant changes in experiences in close relationships score (Table 2).

**TABLE 2:** Comparison between the two studied diagnosis groups according to different scores.

	SCD	Volunteer	*p*	*p* (Bonf)
Score attach secure	(*n* = 16)	(*n* = 39)		
Mean ± SD	75.6 ± 22.2	69.4 ± 29.5	0.70	1.00
Median (min–max)	80 (20–100)	80 (0–100)		
Score insecure avoidant attachment	(*n* = 15)	(*n* = 39)		
Mean ± SD	19 ± 23.9	17.6 ± 22.8	0.95	1.00
Median (min–max)	10 (0–80)	10 (0–87)		
Score insecure anxious‐ambivalent attachment	(*n* = 16)	(*n* = 39)		
Mean ± SD	6.4 ± 8.5	13 ± 19.8	0.62	1.00
Median (min–max)	4 (0–30)	5 (0–67)		
Score disorganizded attachment	(*n* = 15)	(*n* = 39)		
Mean ± SD	0.8 ± 2.6	16.2 ± 22.4	0.002*	0.01*
Median (min–max)	0 (0–10)	5 (0–96)		
Score ECR RA	(*n* = 15)	(*n* = 31)		
Mean ± SD	2.7 ± 0.7	2.7 ± 1.3	0.65	1.00
Median (min–max)	2.8 (1.2–4)	2.4 (1.1–6.5)		
Score ECR RE	(*n* = 15)	(*n* = 31)		
Mean ± SD	2.8 ± 1.1	2.7 ± 1.2	0.80	1.00
Median (min–max)	2.2 (1.4–4.8)	2.5 (1.2–6.7)		

*Note: p*: *p* value for comparing between the studied groups. *p* (Bonf): *p* value after Bonferroni correction.

Abbreviation: SD, standard deviation.

* Statistically significant at *p* ≤ 0.05.

**TABLE 3:** Univariate and multivariate linear regression analysis for the parameters affecting disorganized attachment score (*n* = 54) for total sample.

Predictor	Univariate *p* (*B*, 95% CI)	Multivariate *p* (*B*, 95% CI)
SCD	0.011* (−15.4, −27.1 to −3.7)	0.025* (−14.4, −26.9 to −1.9)

Abbreviations: B, unstandardized coefficients; CI, confidence interval.

* Statistically significant at *p* ≤ 0.05.

## 4 Discussion

To our knowledge, this is the first systematic study on perceived relationship quality in persons affected by SCD. Our findings may improve understanding of couple dynamics, even at the earliest stages of a partner's cognitive impairment. Increased awareness of relationship difficulties as a result of cognitive impairment may facilitate timely couple therapy. In our study, we have investigated possible links between SCD and different attachment styles. Interestingly, disorganized‐insecure attachment scores were significantly lower in SCD patients than in control subjects. This association remained significant after controlling for possible confounders. This could indicate a subjective need for a high degree of structure and security in the personal lives of the couples concerned. However, further studies are needed to substantiate this assumption with data on personality traits and longitudinal psychopathology trajectories in SCD.

While medical history indicated no significant differences between groups in general somatic health status, we did observe an association of SCD with a history of renal disease, cardiovascular disease, and hypercholesterolemia. SCD did not correlate with secure attachment, ambivalent insecure attachment, or avoidant insecure attachment. The results show that our sample (16 SCD patients) tends to have a consistent level of engagement with partners. They show openness in expressing thoughts and feelings related to cognitive difficulties. People with SCD feel comforted and protected by the presence of their partners. They are comfortable depending on their partners and can balance the act of giving and receiving in a couple.

Close relationships in couples in which one partner is affected by cognitive decline have been widely researched [14, 15]. Behavioral changes associated with cognitive disorders often negatively affect the quality of couple relationships [23]. A recent cross‐sectional study focused on married couples in which one spouse received a diagnosis of mild cognitive impairment (MCI) shows that cognitive decline and associated behavioral changes are distressing for spouses and significantly affect marital quality by increasing emotional stress [23]. Consistently, a significant negative impact of cognitive impairment on spousal communication has been reported [24]. A poor premorbid relationship and significantly impaired relationship quality are predisposing factors for perceived strain and depression of the caring partner [25]. As reported by a recent review, which also includes younger‐onset cognitive impairment, cognitive decline is associated with a decrease in relationship quality and self‐esteem, changes in identity, shifts in roles, changes in responsibilities, increasing social isolation, and shifts in relationship quality [12]. Relationship quality may be affected on many levels, including marital cohesion, communication, emotional expression, and increased caretaker burden [14]. Consistently, up to 30% of patients with MCI may suffer from emotional and behavioral symptoms as a consequence of impaired relationship quality [26], including perceived lack of companionship and a feeling of loneliness [27], despite continued physical presence [28]. Our study expands these earlier investigations on individuals affected by SCD, which by definition do not (yet) represent an objectively measurable impairment, but nevertheless are a burdensome disorder on the level of subjective perception. However, early adequate psycho‐educational approaches offered to persons affected by SCD may help couples in modifying behaviors and expectations in a context of an increased risk of a progressive neurodegenerative disease such as AD [9]. Such early therapeutic interventions may help to maintain relationship satisfaction and preserve quality of life [29]. Findings of our research suggest measurable changes in attachment style in couple relationships of persons with SCD. An example of early intervention is emotionally focused couple therapy, which is a systemic approach based on attachment theory [30]. Depending on individual needs, integrative couple therapy could also be applied [31], ideally as part of a coordinated psychotherapeutic treatment intervention aimed at the quality of life of persons with SCD, as well as their families.

*The current study has several limitations*: First, the size and composition of the sample create potential limitations on generalizability to the population of SCD patients. This includes the healthcare‐seeking behavior that led to study participation. No data were collected on the presence of companions at the first visit or on specific motivations for seeking professional advice and diagnostic assessment. Second, reliance on the perspective of only one member of the couple. Third, our study's cross‐sectional design limits conclusions regarding the predictive aspect of the relationships. Fourth, lack of information on personality traits, social and environmental factors, as well as the evolution of the psychopathology of affected patients and their caring spouses.

## 5 Conclusion

The impact of cognitive decline goes beyond memory problems and significantly affects couples' relationships at several levels. Although coping with progressive cognitive decline in diseases such as Alzheimer's is extremely difficult for all concerned, the quality of the relationship, even in its earliest stages, can be an important source of support for the person with Alzheimer's and their spouses. Any opportunity to intervene should be taken as early as possible. The results of our study on persons with SCD help us to better understand changes in relationship quality before the onset of dementia. Further longitudinal studies are needed to better understand the role of personality traits and the progression of individual psychopathology. With this information, specific targeted psychotherapy interventions can then be developed and validated for different levels of cognitive impairment.

## Author Contributions

**Mohamed Eshmawey:** conceptualization, visualization, investigation, methodology, formal analysis, writing, reviewing and editing. **Sonja M. Kagerer:** methodology, data analysis and reviewing. **Federica Ribaldi:** investigation, resources, data curation. **Aïda B. Fall:** visualization, reviewing and editing. **Paul G. Unschuld:** visualization, reviewing, editing and supervision.

## Conflicts of Interest

The authors declare no conflicts of interest.

## Data Availability

The data that support the findings of this study are available from the corresponding author upon reasonable request.
